# Review of Risk Factors, Pathophysiology, Management Principles, and Role of Medications

**DOI:** 10.1155/2024/5456490

**Published:** 2024-04-09

**Authors:** Shreyus Kankanady Shivanand

**Affiliations:** Monash Health, Melbourne, VIC, Australia

## Abstract

This is a case report of one patient experiencing psychotic symptoms in the setting of Charles Bonnet syndrome (CBS). Case description is included, and patient has been deidentified. Patient's consent could not be obtained for the submission of the report. The case report focuses on understanding and formulating key psychological issues addressed in this case. It is important to identify that the absence of psychotic illness is classical in patients presenting with psychotic symptoms in CBS and the role of antipsychotic medication is uncertain. A literature review on the management of CBS guidelines published across the world and summarization of the management approach applicable to this case. Visual hallucination is a perception of a visual stimuli when none exists. CBS is characterized by the presence of complex visual hallucinations experienced by the visually impaired, i.e., in an individual with ocular pathology causing vision loss without having true psychosis or dementia. Furthermore, the person having these experiences has a preserved insight into the unreal nature of the perceptions and the absence of mental disorders. An introduction to the terminology “atypical CBS” or “CBS plus” was done to consider visual hallucinations in individuals with low level of insight in a setting of possible cognitive deficits or other hallucinatory modalities.

## 1. Introduction

Aristotle in his noteworthy work De Anima [1–3] (on the soul), 350 B.C. traditionally identified the five human senses including sense of sight, hearing, taste, smell, and touch; and that there is no other sense beyond these. He describes imagination to be a part of “perception” and that it is different from “thinking.” Aristotle explains the idea of phantasia (imagination) being formed from phaos (light) as vision or sight, the most highly developed sense would not be possible in the absence of light. The interplay of imagination and sensation remains in the organs which guides animals and their actions, and in others, it is “nonexistence could be due to a temporary eclipse by feeling or disease or sleep” [1].

## 2. Case

This case report is of 57-year-old single male, who was admitted to medical unit for management of diabetic ketoacidosis on background of poorly controlled T2DM (insulin dependent). He was referred to CL psychiatry for complaints of worsening visual hallucinations. His presentation of visual hallucination is in the setting of significant longstanding bilateral vision decline and he had been deemed legally blind 3 years ago due to (1) progressive diabetic retinopathy in the right eye and (2) a phthisical left eye postsurgery for retinal detachment 20 years ago post water-skiing accident. A diagnosis of Charles Bonnet syndrome (CBS) was made over 18 months prior to this presentation by the ophthalmologist whom he has been engaging with for the last many years. In addition, he has had numerous diabetic complications over the years including nephropathy, neuropathy (Charcot's arthropathy of right foot), and peripheral vascular disease and has been closely managed by the relevant subspecialists.

While the nature of his visual hallucinations had been progressive, there was a noted acute worsening over the 4 months prior to this current presentation, and it appears to coincide with being made redundant from his job of 30 years due to employer concerns regarding safety of himself and others in the workplace. There has also been a rapid decline in his functioning ability, and he uses a cane when ambulating. While he continued to maintain the view that there was an obvious contradiction evidence of the visual experiences being untrue and had a reasonable inference of the external stimuli, these visual hallucinations were distressing to the extent such that it was affecting his ability to engage in the workplace and relationship with his partner.

### 2.1. Psychopathology

The list below describes the psychopathology of his presentation:

- Conviction that he is seeing the manager of the previous workplace at home.
- Seeing distinct shapes which he believes are figures of people from his workplace coming into the house and working around him.
- Misinterpreting stimuli around him particularly figures of people in the house and wondering if his partner might be cheating on him on one occasion. This was a brief thought and he was able to distinguish this from reality and discuss it with his partner.
- Visions of things around the house being moved and shapes he believes to be his work colleagues, standing over him whilst working, etc., and bullying him by creating chaos in his work environment.
- Believes belongings and other things including layout of the house being distorted and moved around.
- Perception of things being moved particularly food in the fridge and his medications.

There is no known family history of mental health issues, dementing illness, vision impairments, or other significant medical comorbidities. This gentleman has been a nonsmoker all his life, with consumption of 1–2 standard drinks of alcohol on special occasions including Christmas and his birthday. Other medical history included stable hypertension managed with antihypertensive medication therapy.

### 2.2. Clinical Findings and Diagnostic Assessments

Systemic examination and routine blood investigations were unremarkable. Neurological examination was unremarkable. A noncontrast CT brain was done on admission. This was comparable with a similar study done 9 years earlier in the context of a head injury postfall. The ventricles, sulci, and basal cisterns appear normal, the gray–white matter differentiation is preserved and no acute intra- or extra-axial abnormalities were seen. There was no evidence of an acute haemorrhage or infarction. The current noncontrast CT brain showed that the ventricular and sulci were mildly prominent for patient's age suggesting generalized atrophy and this has developed since the previous CT. There was no haemorrhage or acute cortical infarction. Periventricular low attenuation was noted and suggestive of chronic small vessel ischaemic change. A cognitive assessment was performed with MOCA-blind where he scored 21 (scored 2 out of 3 for attention assessment in Serial-7s), this was not indicative of a cognitive deficit.

## 3. History

De Morsier identified the noteworthy observation of Charles Bonnet, a renowned Genoese Philosopher in the eighteenth century who described the strange visual perceptual images experienced by his otherwise cognitively and intellectually intact, yet visually impaired grandfather Magistrate Charles Lullin [4] who suffered from cataract and underwent surgery in both eyes. Bonnet [5] in his first scientific documentation described Lullin's experiences of visions of silent men, women, birds, carriages, and buildings as “fictions” of his mind. He writes about the innocence of the mind that is at play. This description of Lullin's experiences was documented as seeing objects that made different movements, changing tapestry of his apartment, etc. Later in his life, Charles Bonnet suffered visual deterioration complicated by visual perceptions, hence lent his name to the term [6] CBS.

The images and objects in various case reports are described as being simple or complex visual hallucinations. These include simple vivid, abstract, or complex including geometrical patterns, moving objects to complex images of people and objects.

## 4. Diagnostic Criteria for Charles Bonnet Syndrome

Gold and Rabins' definition of CBS [1] is the most widely used in making a diagnosis in the absence of an official diagnostic criteria. It includes:

- at least one complex visual hallucination within the past 4 weeks,
- a period between the first and the last hallucination exceeding 4 weeks,
- full or partial retention of insight into the unreal nature of the hallucinations,
- absence of hallucinations in other sensory modalities, and
- absence of delusions.

However, the above criteria as proposed by Teunisse et al. [1] do not include visual impairment as a component for diagnosis of CBS which contrasts with clinical practice of ophthalmologists who consider visual impairment as a main criterion to describe and diagnose CBS. Subsequently, the revision of the International Classification of Diseases [7], by the World Health Organization in 2018, has allocated a specific diagnose code for CBS, i.e., 9D56. Based on this definition, the principal diagnostic criteria include:

- the presence of complex visual hallucinations,
- partial or complete loss of vision, and
- the absence of mental or behavioral disorders.

## 5. Aetiologies of Visual Hallucinations

Cases of CBS are considered rare presentations. However, there has been a spike in the number of reported cases, particularly given the unusual presentation of visual hallucination in subpopulations including geriatric populations presenting with comorbid cognitive difficulties, vascular and cerebral pathologies including cerebrovascular diseases (dementia) [2], stroke; comorbid vision disorders [8] (such as, age-related macular degeneration, glaucoma, and cataracts):

- Neurological disorders.
- Intracranial pathology.
- Psychiatric disorders.
- Dementing illness.
- Substance use related.
- Intoxication/withdrawal.
- Metabolic disorders.
- Complications secondary to ocular pathology.

## 6. Pathophysiology

The strongest risk factors identified for CBS are bilateral visual impairment, declining visual acuity, social isolation, and sensory deprivation. The genesis of visual hallucination is explained by Asad and Shapiro as factors that could be derived from a psycho-biochemical model due to the conductance disturbance in the neurotransmitters, psycho-physiological model due to a structural abnormality in the brain, and psychodynamic model that connects conscious to the unconscious mind.

The visual cortex located in the occipital lobe of the brain plays a role in the processing of visual information that is received by the eye through the lateral geniculate nucleus in the thalamus. The primary visual cortex receives the sensory inputs from the thalamus and the extrastriate cortex situated next to the primary visual cortex is sensitive to motion [8]. Therefore, the generation of visual hallucinations can be explained by the deafferentiation theory that is most widely accepted to explain CBS. The deafferentiation is explained as an alteration in the receptive fields in the visual cortex [9] due to the loss of visual input into the brain, resulting in spontaneous neuronal discharge in the visual cortex, increasing excitability. Thereby, resulting the release of an abnormal perceptions, visual hallucinations. This can explain the absence of visual release hallucinations in people who are born blind due to its association with the increase in cortical activity following sensory deprivations.

Some acquired visual defects following surgical alterations can also result in transient visual hallucination. This might also explain the hypothesis that these visual hallucinations are likely to be generated due to direct damage to the visual pathways. Other pathologies that can cause direct damage to the visual system include cerebrovascular accidents, dementing illness, age-related visual problems like macular degeneration, cataract, and glaucoma. Visual loss can occur in about 25% of patients with temporal arteritis which occurs due to the lack of oxygenated blood supply to the eye and optic nerve. Brain tumors like meningioma are likely to damage the optic nerves and cause blindness.

Maintenance of arousal in conditions like insomnia, sleep-related disorders, semicomatose phase, and drowsiness can be suggestive of the role of reticular activating system in the maintenance of arousal that can be associated with visual hallucinations. Acute lesions in the brain involving cerebrovascular accidents, strokes, and lesions in the brain stem have led to visual hallucinations, i.e., peduncular hallucinosis [10]. These are described as visual, chromatic, detailed, and often moving (Lilliputian) objects in the whole visual field in an individual with intact visual acuity and visual fields.

## 7. Management Principles

Despite the absence of evidence of the role of psychotropic medication, there are a variety of valid treatment options and rehabilitation focused approach aiming for visual and social rehabilitation to provide support and psychoeducation, there is hope for community reintegration and living a productive life [11].

Education and supportive therapy play a vital role in the recovery of these patients. Social support enhancement and working closely with patients and their carers guides to improve the quality of life. Various methods of visual modifications have been discussed and trailed which have been reported to be helping in reducing the durations of hallucinations. Illumination modification methods [12] that have been found beneficial include programed blinking, which is a process of intermittent closing and opening of the eyes. Modification of the surroundings including increasing illumination, particularly during the later hours of the day, and encouraging social and interpersonal contact during these hours can be helpful. Stress and emotions are likely to worsen the perceptual disturbances, hence a low stimulus environment, reducing social isolation might be able to enhance concentration and thinking thereby mitigate the heightened arousal from these strange experiences. Visual aid including the use of optical devices, illumination enhancement, use of spectacles, eye shields to prevent triggers from bright environments, and provision of full-day illumination in the home environment can help improve the sensory profile [13].

There are a few case reports studying visual hallucinations in the visually impaired populations published so far. Although they have not identified a universally effective pharmacotherapy for CBS. There are no known double-blinded, placebo-controlled, and clinical trials that have been completed to study the efficacy of pharmacologic therapy for CBS. Some case studies have identified that SSRI and NARI may be well-tolerated and effective in the resolution of these secondary visual hallucinations. This could be explained by its association of serotonergic pathways in the pathogenesis of visual hallucinations [14].

Correlation between successful use of cholinergic agents for the treatment of visual hallucinations associated with Alzheimer's disease [15] and DLB [16] suggests the likely dysfunction of cholinergic neurons may be an important factor in typical CBS.

Risperidone, a serotonin–dopamine antagonist, is commonly used in the treatment of dementia-related hallucinations [17], and melperone [18] was reported to be effective in some case reports. Melperone is from butyrophenone group and can be considered safe due to its moderate cataleptic features, low affinity for D2 receptors, and its antipsychotic effects due to its influence on the serotonergic and limbic dopaminergic systems in combination with its antiadrenergic properties. A report suggests that occipital transcranial magnetic stimulation may be effective [19].

### 7.1. Therapeutic Intervention in This Case

A trial of low dose of oral paliperidone due to its safe profile in hyperglycaemic patients was found effective in partial resolution of visual hallucination and ameliorated the distress caused by these strange experiences. Psychoeducation was provided to the patient reiterating the unreal nature of these experiences and reassurance of his sanity was comforting for the patient. This was essential in his recovery with focus on reinforcement and education regarding the differentiation of psychotic symptoms from reality and encouraging him to be mindful of his actions in view of his disability. Progressively in the medium term such intervention with supportive psychotherapy would be beneficial to increase attention to sensory deficits, emotional overload due to these deficits, understanding and acceptance of the life challenges and the attribution of perception. Relaxation manoeuvres, stress reduction techniques including mindfulness, yoga could be considered without the need for extensive environmental modification. This could also be beneficial in improving self-awareness and can facilitate consideration to participate in community programs.

## 8. Discussion

In this case, it is difficult to ascertain that the psychotic experiences are purely from CBS due to the association of other comorbidities including cognitive deficits given the age of the patient. Longstanding history of poorly controlled blood sugar levels from an underlying diabetic illness might predispose him to early and rapid progression of vascular disease, hence further heightens risk of ocular pathology. An evolving multimodal etiological theory can therefore better explain the comorbid vascular, cerebral, and ocular model contributing to his presenting visual impairment and its related consequences, i.e., visual hallucination. However, cognitive deficits, intracranial pathological, or other organic causes were ruled out at the time of establishing diagnosis of CBS.

A thorough psychiatric assessment was conducted and pervasive mood symptoms including depressive illness, hypomania, manic disorder, and psychotic illness were ruled out. Despite the distress from his experiences, he had a good insight that the visual experiences were not real. This intact cognition with a preserved insight regarding the visual hallucinations in the absence of psychological conditions is evidence to diagnosis of CBS.

There appear to be numerous biopsychosocial factors affecting the perception secondary to his level of distress. In one spectrum, he has been a high functioning, independent, and confident individual. However, the adversities of physical ailments seem to have impacted his self-morale resulting in difficulty in coping with the loss. The poor understanding of his environmental situations and anticipation of the response of people in his immediate surroundings is likely to have compounded to his disability. These factors might have led to the development and maintenance of his difficulties and their relationship with the psychotic phenomena. A limited understanding of psychiatric illness per se and in current circumstances, its relation to his own experiences due to poor visual ability might contribute to worsening of his coping ability. An alternative theory could be based on the stigma of mental illness that is possibly a staunch expression of communal acceptance which might have further exacerbated his difficulties and struggle with the disability due to blindness.

There is likelihood that the most challenging emotions in times like this is a narcissistic injury. As a high-achieving person, I can only imagine how unsuccessful results or life adversities can be uncomfortable because it is outside of the usual pathways one treads. Injustice that he sustained at work and the distress is likely to have affected by obscuring, minimizing, and denying the powerful links between his deficits and psychotic element. Stress of perceived rejection from his partner as they did not seem to be coping with the worsening distress and behaviors might have heightened the intensity of perceptual disturbances. The theory of mentalization describes the inability to psychologize physical symptoms as suggested by Mattis in “Basic processes” [17, 20] that an affect is a predisposition to a certain type of reaction to external stimuli. This is by virtue of the conscious component seen as one's own feelings or which remains unconscious expresses as physically or somatically or both.

The possible differential diagnosis [13] that were considered primarily are delirium secondary to infective or inflammatory process, delirium secondary to complications of diabetes mellitus (i.e., hypoglycaemia, hypoglycaemic seizures, diabetic ketoacidosis, urinary tract infection, acute kidney injury, or hepatic disease), metabolic encephalopathy, uraemia endocrine disturbances, intracranial pathology, intracranial infection (meningitis vs. encephalitis) in light of worsening symptoms of perceptual disturbances at night with mildly elevated inflammatory markers, alcohol hallucinosis, photospies are commonly associated with epilepsies as aura-like phenomena, an acute onset of visual hallucinations is commonly associated with homonymous hemianopia or optical nerve damage, complex hallucinations are experienced after cerebral lesions or after visual field defects and medication induced [21].

The other differential diagnosis to be considered are head trauma, migraine, intracranial tumors, and substance-induced hallucinosis, sleep deprivation, dehydration, narcolepsy–cataplexy syndrome, prolonged isolation, stress, or fatigue-induced reaction [7]. However, my provisional diagnosis is psychotic episode secondary to medical condition, i.e., CBS, presenting with perceptual disturbances and delusional elements due to sensory deficit.

The aim of my involvement in the care of this patient in a psychiatric role within a medical setting was to recognize his immediate needs, careful screening for organic pathology, ophthalmologic, and neuropsychiatric interventions [2] that need to be catered to. The likelihood of medical complications resulting in altered sensorium cannot be underestimated and thereby warrants a thorough systemic work-up. Preliminary haematological and biochemical assay, inflammatory markers to rule out an underlying delirium would be essential. He has suffered previous hypoglycaemic seizures and hence EEG was considered, and seizure activity was ruled out.

## 9. Conclusion

In conclusion, one cannot emphasize enough the importance of education to the treating teams and other care providers of patients with CBS are aware that the psychotic symptoms that the patient is experiencing are not associated with mental illness. It is not uncommon for such patients to go through a prolonged period of suffering because of lack of awareness of medical specialists to identify the association between the two [2].

The determinants of a positive prognostic indicators that might be beneficial in working toward recovery are lack of genetic predisposition to dementing illness and psychiatric illness, absence of substance use history, reasonable social support, and willingness to focus on recovery through social interventions, reasonably preserved cognitive ability.

Visual Impairment is a burden. One can only imagine what might be a subjective experience to see strange unreal objects or images which contrast with reality. The dilemma and fear of bearing a mad brain do heighten the stress for these vulnerable individuals. Hence, despite the unreal nature of these images and experiences, they can cause significant distress and be perceived as disturbing by these patients. Psychiatric and psychological implications of these experiences could be recognized and better understood, hence a review of mental state to follow-up on the implications and evolution of psychiatric comorbidities through the course of the illness could be evaluated, monitored, and managed better. Lastly, albeit the suffering, the patient was able to recognize the unreal nature of his perceptual disturbance with reassurance that lack of mental illness would help him recover in a safe manner despite the disability.

## Data Availability

Shreyus Kankanady Shivanand can make the data available on request and can be contacted via email at drshreyus@gmail.com or shreyus.shivanand@monashhealth.org.
